# Network Pharmacology Analysis and Experimental Verification on Antiangiogenesis Mechanism of *Hedyotis diffusa* Willd in Liver Cancer

**DOI:** 10.1155/2023/1416841

**Published:** 2023-01-07

**Authors:** Hongyan Wu, Lihu Zhang, Chuntao Wang, Fang Li, Liang Qi, Linxia Xiao, Mingguang Zhang, Hu Zhang, Guozhe Zhang, Yiyu Qin

**Affiliations:** ^1^Institute of Biomedical Technology, Jiangsu Vocational College of Medicine, Yancheng, China; ^2^School of Pharmacology, Jiangsu Vocational College of Medicine, Yancheng, China; ^3^Department of Basic Medicine, Jiangsu Vocational College of Medicine, Yancheng, China

## Abstract

**Purpose:**

*Hedyotis diffusa* Willd (HDW) is one of the most well-known herbs used in the therapy of cancer. However, the potential mechanisms of its antiangiogenic effects have not been fully explored. Here, we applied a network pharmacology approach to explore the potential mechanisms of HDW against liver cancer angiogenesis (LCA) and used a mouse orthotopic liver cancer model for experimental verification accordingly.

**Methods:**

The effective components, primary active compounds, and possible targets in the therapy of LCA were predicted using network pharmacology and bioinformatics. In vivo testing of the pharmacodynamic foundation of HDW in the treatment of LCA was performed. Hepa1-6 cells were implanted in C57BL/6 mice to establish an orthotopic liver cancer model to evaluate the antitumor and antiangiogenesis effects of the drug. Furthermore, protein levels were evaluated by western blotting, immunofluorescence, and immunohistochemistry.

**Results:**

We firstly confirmed the therapeutic effect of HDW on LCA and subsequently screened 7 active compounds from HDW according to their pharmacokinetic properties. Network analysis and enrichment analysis indicated that these compounds exhibit antiangiogenic effect by acting on multiple targets and thereby regulating multiple pathways mainly involved in Akt1, IL-6, IL-1*β*, IL-17, hypoxia inducible factor-1*α* (HIF-1*α*), and tumor necrosis factor-*α* (TNF-*α*). Importantly, we preliminarily verified the results of the network pharmacology analysis in vivo.

**Conclusion:**

Collectively, our work initially explored the therapeutic mechanism of HDW on tumor angiogenesis, which lays an experimental reference for further exploring its pharmacological action and its clinical application.

## 1. Introduction

Angiogenesis is an essential factor in tumor progression. Tumor cells can get the nutrients and oxygen they need for sustained angiogenesis. Angiogenesis plays a vital role in promoting tumour growth and metastasis [1]. As one of the most common malignant tumors and the fourth leading cause of cancer-related deaths worldwide, liver cancer is characterized by an abundant blood supply and microvessels [2–4]. Microvessel density has been reported to be a significant predictor of death and closely correlated with tumor stage in liver cancer [4]. Therefore, angiogenesis has become a therapeutic target, and new agents of antiangiogenic therapy are urgently required for liver cancer treatment [5].

Increasing evidence has shown that Chinese medicinal active compounds offer unique advantages due to the synergy effect of multicompounds, multitargets, and minor adverse reactions in the antitumor aspect [6]. As a traditional medicinal plant, *Hedyotis diffusa* Willd (HDW) has been recorded for thousands of years in clinical applications. It is widely used for heat clearing, detoxification, and removal of blood stasis [7]. Moreover, HDW has long been used as an important component in a variety of Chinese medicine formulas to treat various types of cancer [8–12]. Existing research has reported its noticeable antitumor effect on hepatocellular carcinoma (HCC) [10]. However, the effect of HDW on LCA and the potential mechanism of its antiangiogenic effect have not yet been explored.

**Figure 1:**
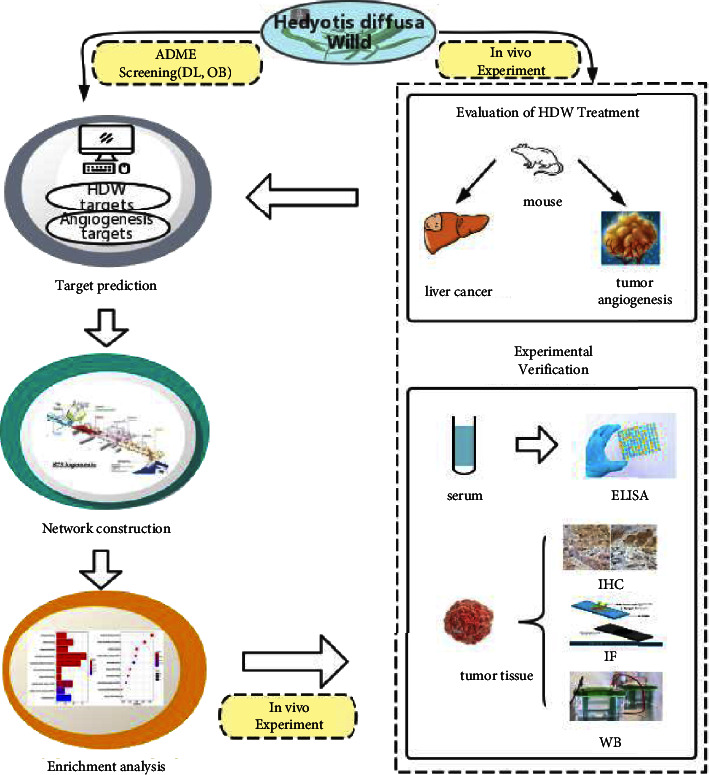
Workflow for network pharmacology approach in liver cancer angiogenesis treatment with HDW. ADME: absorption, distribution, metabolism, and excretion; DL: drug-likeness; OB: oral bioavailability.

As an emerging subject, network pharmacology can comprehensively and systematically clarify the mechanism and targets of Chinese medicinal active compounds, which are fully compatible with the characteristics of traditional Chinese medicine (TCM) in terms of multicomponent, multitarget, and multipathway properties [13, 14]. In this study, we initially started our analysis by confirming the treatment effect of HDW using the mouse orthotopic liver cancer model. Then, we applied the network pharmacology approach to explore the pharmacological mechanisms of HDW as an antiangiogenic therapy for HCC. Subsequently, we validated the potential targets and pathways of HDW as a therapy against LCA using molecular biological methods (Figure 1). Therefore, this study aims to provide a foundation for future clinical applications on the effectiveness of HDW in the treatment of liver cancer.

## 2. Materials and Methods

### 2.1. Drugs and Reagents

*Hedyotis diffusa* Willd injection was purchased from Keyuan Pharmaceutical Co., Ltd. (specified as 2 ml each, Fengyang, Anhui). The following primary antibodies were used in this study: Akt1, p-Akt1, mTOR, p-mTOR, STAT3, and p-STAT3 (Cell Signaling Technology, Danvers, MA, USA); CD31, HIF-1*α*, and GAPDH (Abcam, Cambridge, UK); and *α-*SMA (Absin, Shanghai, China). The fluorescein isothiocyanate (FITC)-conjugated secondary antibodies and all horseradish peroxidase-conjugated secondary antibodies were obtained from Proteintech (Wuhan, China). The mouse ELISA kit was purchased from PeproTech Inc. (Rocky Hill, USA). Hepa1-6 tumor cells were obtained from iCell Bioscience Inc. (Shanghai, China) and cultured according to the manufacturer's instructions.

### 2.2. HDW Bioactive Compound Screening and Target Identification

All chemical compounds of HDW were obtained from the Traditional Chinese Medicine Systems Pharmacology (TCMSP) database [15]. After the data were gathered, oral bioavailability (OB) and drug-likeness (DL) were utilised to screen the bioactive components of HDW. Only compounds with OB ≥ 30% and DL ≥ 0.18 were chosen and identified as potential bioactive compounds for further investigation. These selected compounds were subsequently used for target identification and compound-target network establishment. Then, we utilised the UniProt database (https://www.uniprot.org/) to calibrate the gene names of all targets, using humans as the chosen species [16].

### 2.3. Probing LCA Targets and Venn Diagram Establishment

The LCA targets were obtained from two different sources [17, 18]: (1) the GeneCards database (https://www.genecards.org/) and (2) the NCBI gene database (https://www.ncbi.nlm.nih.gov/). Our investigation searched these human illness target databases for targets associated with LCA using the keywords “liver cancer angiogenesis,” “hepatocellular carcinoma angiogenesis,” and the species “homo sapiens.” After merging and deleting the genes in the two databases, the target genes associated with LCA were obtained; common targets associated with LCA and potential targets of bioactive substances were chosen as HDW's targets against LCA. A Venn diagram of the intersection between the target of the drug and the disease was established using Venny 2.1.

### 2.4. Construction of Protein Interaction Network

The Search Tool for the Retrieval of Interacting Genes (STRING, https://string-db.org/cgi/input.pl) was used to collect possible protein-protein interactions (PPI) by uploading 116 common targets related to LCA and putative targets of active compounds. Species were limited to “homo sapiens” with a confidence score >0.4. Then, we imported the database from STRING into Cytoscape (version 3.7.1) to construct a PPI network for further analysis [19].

### 2.5. Enrichment Analysis

Enrichment analysis included the Gene Ontology (GO) Enrichment Analysis and the Kyoto Encyclopedia of Genes and Genomes (KEGG) Pathway Enrichment Analysis. (1) GO enrichment analysis was performed by using the ClusterProfiler package of R4.0.3 and mainly included biological process (BP), molecular function (MF), and cellular component (CC). GO terms with the STRING database-corrected *p* value ≤0.05 were retained for the construction of the clustering network [20]. (2) KEGG pathway enrichment was performed by using the ClusterProfiler package of R4.0.3, and KEGG terms with STRING database corrected-*p* value ≤0.05 were retained for constructing a target-pathway network [21].

### 2.6. Mouse Orthotopic Liver Cancer Model Construction and Drug Treatment

**Figure 2:**
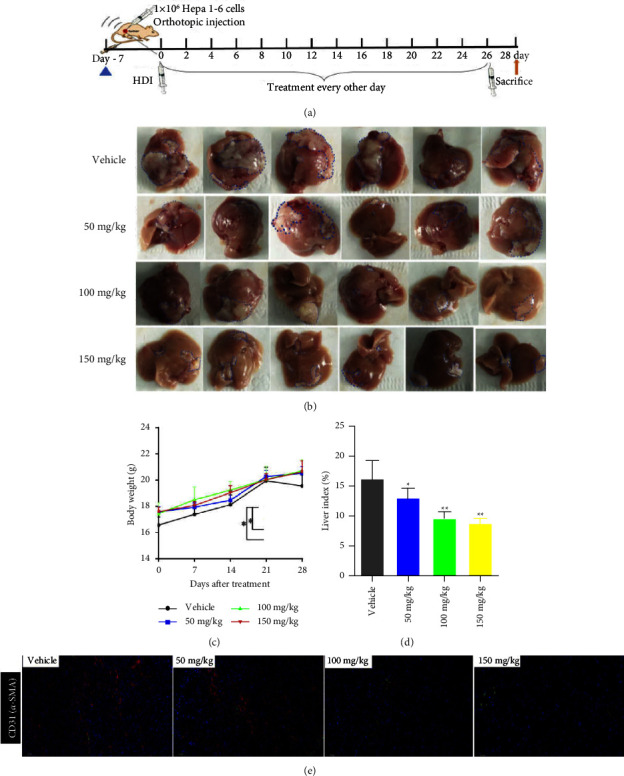
Treatment effect of HDW on liver cancer growth and angiogenesis in the orthotopic mouse models. (a) The schedule of orthotopic liver cancer model treatment and imaging. (b) C57BL/6 mice with orthotopic Hepa1-6 tumor burden were treated with HDI intraperitoneally (50, 100, or 150 mg/kg) every other day (*n* = 6 per group). Representative photographs of liver tumors after the indicated treatments; the blue dotted lines indicate the tumor regions. (c) Body weight curve in vehicle and HDW-treated group. (d) Liver index in vehicle and HDW-treated group. (e) Immunofluorescence staining examination of CD31 and *α*-SMA in tumor sections from vehicle or HDW-treated group. All panels are of 200x magnification. Scale bar = 50 *μ*m (^*∗*^*P*  <  0.05 and ^*∗∗*^*P*  <  0.01 vs. vehicle).

C57BL/6 mice (male, 4∼6 weeks) were purchased from Beijing SiPeiFu Biotechnology Co., Ltd. and fed in a pathogen-free vivarium under standard conditions. All animal experimental protocols and principles were approved by the Animal Care and Use Committee of Jiangsu Vocational College of Medicine. To establish an orthotopic liver cancer model, 1 × 10^6^ Hepa1-6 tumor cells were suspended in 50 *μ*L Dulbecco's Modified Eagle Medium (DMEM) (containing 20% Matrigel) and orthotopically injected into the liver of C57BL/6 mice under anesthesia with tribromoethanol (240 mg/kg, Sigma, Massachusetts, USA) [22]. After 7 days, tumor-bearing mice were randomized into a vehicle and different treatment groups (1 group, 6 mice): (1) The vehicle group mice were injected with normal saline. (2) The treatment groups were intraperitoneally injected with HDW injection (50 mg/kg, 100 mg/kg, or 150 mg/kg) every other day 14 times, and the body weight was monitored every 7 days (Figure 2(a)).

### 2.7. Immunofluorescence (IF) Staining and Immunohistochemistry (IHC)

Immunofluorescence (IF) staining has been previously described [23]. Mouse liver cancer tissue samples were immediately frozen in OCT compound or fixed in 4% PFA overnight at 4°C, dehydrated, and embedded in paraffin. Samples were blocked with 5% goat serum in PBST (0.3% Triton X-100 in PBS) and then incubated for 1 h at room temperature with the following primary antibodies: anti-CD31 (1 : 200) and anti-*α*-SMA (1 : 500). Following washing several times, the samples were incubated for 1 h at room temperature with secondary antibodies (diluted 1 : 200) conjugated with FITC and 4,6-diamidino-2-phenylindole (DAPI) for counterstaining nuclei. The coverslips were mounted on glass slides, and the immunofluorescence staining was visualized and photographed using a Zeiss inverted fluorescence microscope.

An immunohistochemical assay was conducted according to our previous study with slight modifications [24]. In brief, sections after antigen retrieval were incubated overnight with primary antibodies against HIF-1*α* (1 : 200) at 4°C, biotin-conjugated secondary antibodies, and horseradish peroxidase-conjugated streptavidin successively. Then, the sections were stained with diaminobenzidine and counterstained with hematoxylin. The staining density was assessed as in our previous study [24]. The details are as follows: negative results of no or weak staining: invisible or light brown staining in <20% of the cell populations; positive results of moderate or strong staining: brown or dark brown staining in >20% of the populations.

### 2.8. Enzyme-Linked Immunosorbent Assay (ELISA)

The inflammatory factor levels in serum were detected with enzyme-linked immunosorbent assay kits (PeproTech, Rocky Hill, NJ) according to the manufacturer's instructions.

### 2.9. Western Blotting (WB)

The western blotting assay was done as previously described in our study [25]. In brief, total lysates were prepared from tumor tissue. The protein concentration was calculated by the BCA (bicinchoninic acid) Protein Assay Kit (Beyotime, Shanghai, China). Then, the protein samples experienced sodium dodecyl sulphate-polyacrylamide gel electrophoresis (SDS-PAGE) and membrane transfer. The membrane was blocked overnight, then the primary antibodies (Akt1, p-Akt1, mTOR, p-mTOR, STAT3, p-STAT3, and GAPDH (1 : 2000); HIF-1*α* (1 : 1000)) and secondary antibodies (1 : 5000) were added for electrochemiluminescence (ECL) coloration, and the image was semiquantitatively analyzed with alpha SP image analysis software. GAPDH was used as an invariant control for equal loading of total proteins. The proposed blots are representative of three independent experiments.

### 2.10. Statistical Analysis

Data were presented as means ± standard deviation (SD). The statistical significance of the difference was evaluated by the unpaired Student's *t*-test or one-way ANOVA, as appropriate. Values of *p*  <  0.05 were considered statistically significant.

## 3. Results

### 3.1. HDW Suppressed Tumor Growth and Angiogenesis in the Orthotopic Mouse Model

To investigate the treatment effect of HDW on LCA, we established a mouse orthotopic liver cancer model and subsequently evaluated the attenuation effect of HDW on tumor growth and angiogenesis. The in vivo experimental schema is depicted in Figure 2(a).

As shown in Figure 2(b), HDW induced the most marked regression in the orthotopic tumors. In addition, we observed a significant decrease in angiogenesis with IF staining on the morphology of tumor tissue after HDW treatment, indicating its potential effect on attenuating tumor angiogenesis in HCC (Figure 2(e)). Meanwhile, the mouse's body weight was remarkedly declined in the vehicle group rather than in the HDW-treated group on day 28 (Figure 2(c)). In addition, the liver index (liver-to-body weight) was also significantly increased in the HDW group and this effect tended to be dose-dependent (Figure 2(d)). The above results manifest that HDW suppressed liver cancer growth and angiogenesis in mouse orthotopic models.

### 3.2. Active Compounds of HD Screening

TCMSP yielded a total of 7 active compounds of HDW after ADME screening with OB ≥ 30% and DL ≥ 0.18. All 7 compounds were validated as promising bioactive molecules for future research. Detailed information is presented in Table 1.

### 3.3. Common Target Analysis of HDW and LCA

**Figure 3:**
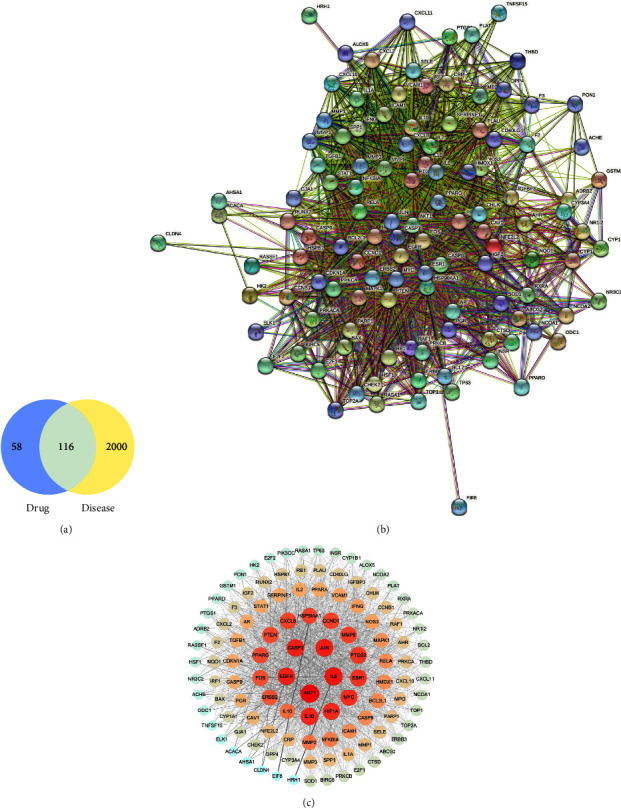
HDW and disease target identification and protein-protein interaction (PPI) network establishment. (a) Venn diagram of HDW gene and target of drug and liver cancer angiogenesis intersection. (b) The original PPI data generated from the STRING database showing the detailed interactions of the targets. (c) The PPI network constructed using Cytoscape (version 3.7.1); the average degree was 34.3, the node color and size are adjusted according to the degree value, the redder the color, the greater the degree; the thickness of the line expresses the size of edge betweenness.

174 putative targets linked to 7 compounds of HDW were collected. A total of 2116 genes related to LCA were obtained. Through Venny analysis, 116 common targets of HDW in the treatment of LCA were obtained (Figure 3(a)).

### 3.4. Common Target Network Analysis and PPI Network of Common Targets

116 common targets were uploaded to the STRING database, and the PPI network was generated with the following conditions: combined score (≥0.4) and species limited to “homo sapiens.” After that, the PPI network was established, as shown in Figure 3(b). The network graph has 116 nodes and 1,992 edges, and the average node degree is 34.3. In addition, we then imported the PPI results into Cytoscape for topology analysis of PPI networks. A closer node to the center and the darker color represent the protein that has a greater degree in this network; then, the top seven core targets were extracted, which were successively Akt1, IL6, IL-1*β*, HIF-1*α*, EGFR, JUN, and CASP3 (Figure 3(c)).

### 3.5. Enrichment Analysis of GO and KEGG Pathway Analysis

**Figure 4:**
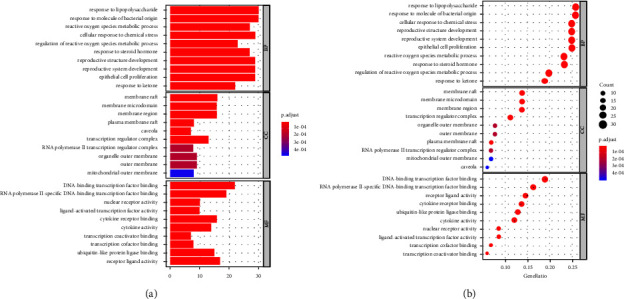
Gene enrichment (GO) analysis for the 7 shared HDW compound targets/liver cancer angiogenesis-related targets. The color represents the different adjusted *P* values (*<*0.05), while the size of the circle represents the count. The top 10 significant GO terms (BP, MF, and CC) are chosen according to the enrichment score, as shown in (a) the bar chart of GO enrichment analysis and (b) the bubble diagram of GO enrichment analysis. BP, biological process; CC, cell compound; MF, molecular function.

**Figure 5:**
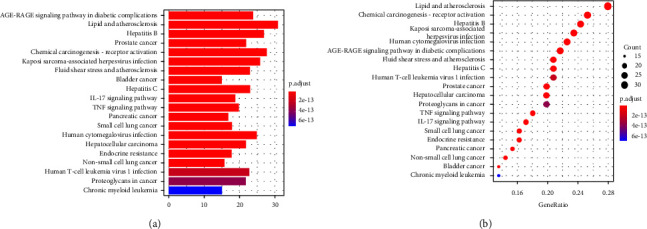
KEGG enrichment analysis for the 7 shared HDW compound targets/liver cancer angiogenesis related targets. The color represents the different adjusted *P* value *<*0.05, while the size of circle represents the count. 20 possible signalling pathways are listed in (a) the bar chart of KEGG pathway enrichment analysis and (b) the bubble diagram of KEGG pathway enrichment analysis.

To further explore the underlying mechanisms of HDW against LCA, we performed GO enrichment and KEGG pathway analysis, executed using the DAVID database (https://david.ncifcrf.gov/home.jsp). GO analysis includes biological processes (BP), cellular components (CC), and molecular functions (MF), which together describe the functions of gene products. The top 10 significantly enriched GO targets are presented (adjusted *p* value <0.05) in Figure 4. As shown in Figure 5, the KEGG pathway enrichment analysis of HDW against LCA mainly involves the TNF-*α* signalling pathway, IL-17 signalling pathway, and so on.

### 3.6. Experimental Validation of the Effect of HDW on Multipathway In Vivo

**Figure 6:**
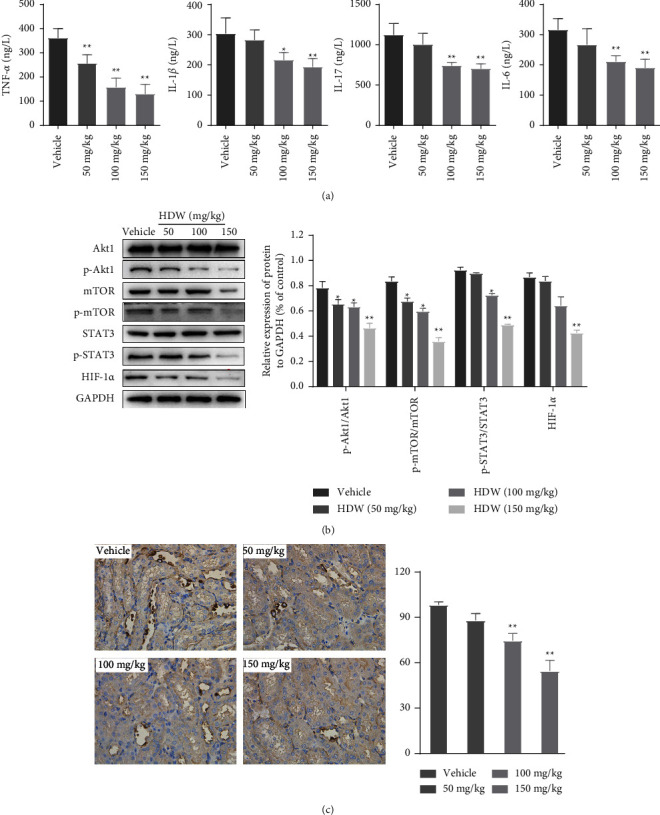
In vivo validation of multipathway effect of HDW. (a) The levels of serum inflammatory factors IL-6, IL-1*β*, IL-17, and TNF-*α* were measured in the orthotopic liver cancer mouse models to four different groups. (b) The effect of HDW on the expression of p-Akt1/Akt1, p-mTOR/mTOR, and p-STAT3/STAT3. The tumor lysates were harvested, and phosphorylation of indicated proteins was detected by western blotting. GAPDH was used as the invariant control. (c) Immunohistochemical staining examination of HIF-1*α* in tumor sections from the vehicle or HDW-treated groups. All panels are of the 200x magnification. Scale bar = 50 *μ*m (^*∗*^*P*  <  0.05 and ^*∗∗*^*P*  <  0.01 vs. vehicle).

To further better validate the putative multipathway mechanisms of the HDW effect according to the results of network pharmacological prediction. We have further studied the effects of HDW on the production of inflammatory factors and the protein expression of multiple key signalling pathways in vivo experiments. As a result, ELISA analyses showed that the inflammatory factors in the serum were decreased in the HDW treated group, including IL-6, IL-1*β*, IL-17, and TNF-*α*, as predicted above (Figure 6(a)). Moreover, western blot analyses showed that HDW treatment decreased the rates of p-Akt1/Akt1, p-mTOR/mTOR, and p-STAT3/STAT3 (Figure 6(b)). All the above results showed significant differences at doses greater than 100 mg/kg. As a well-known core molecule of tumor angiogenesis potential, HIF-1*α* was also detected by WB analyses and IHC staining. As shown in Figures 6(b) and 6(c), both WB and IHC analyses revealed that HDW could downregulate the protein expression level of HIF-1*α* compared with the vehicle group. Collectively, these results confirmed our previous putative consequence that HDW exerted a multipathway therapeutic effect on LCA through regulation of anti-inflammatory processes and key signalling pathways such as those of Akt1, STAT3, and the core molecule HIF-1a.

## 4. Discussion

HDW is one of the most effective medications for clearing heat and detoxicating, with the effects of dispersing the mass, relieving pain, promoting urination, and removing dampness [7, 26]. Modern research has shown that HDW has anticancer properties via multiple pathways and is a commonly used TCM in clinical cancer treatment [7, 10, 11]. In light of previous reports, HDW can suppress tumor angiogenesis [27, 28], but the exact mechanism is unknown.

Up to now, the research studies on the network pharmacology analysis of HDW are scarce [9, 12]. This study uses a network pharmacology approach to select 7 active compounds and 174 related targets and then extracts 116 targets from the intersection of active compounds and liver cancer angiogenesis to build a “target-component” visualisation network. The result about the predicted compounds in the HDW extract in this study is consistent with the previous literature [9, 12]. 2,3-Dimethoxy-6-methyanthraquinone (DMQ), 2-methoxy-3-methyl-9,10-anthraquinone (MMA), (4aS,6aR,6aS,6bR,8aR,10R,12aR,14bS)-10-hydroxy-2,2,6a,6b,9,9,12a-heptamethyl-1,3,4,5,6,6a,7,8,8a,10,11,12,13,14b-tetradecahydropicene-4a-carboxylic acid (HHTCA), poriferasterol, stigmasterol, *β*-sitosterol, and quercetin are the active compounds, and the core targets related to tumor angiogenesis include Akt1, IL6, IL-1*β*, and HIF-1*α*.

Therefore, Akt1 is a serine/threonine-specific protein kinase, a member of the protein kinase B family, and a crucial protein in the PI3K/Akt signalling pathway [29]. As an essential mediator of angiogenesis and metabolism, Akt1 exists widely in vascular endothelial and smooth muscle cells. HIF-1*α* is a core molecule that induces tumor angiogenesis and can directly regulate vascular endothelial growth factor (VEGF) expression at the gene level [30] or indirectly regulate the expression of angiogenic factors such as epidermal growth factor receptor (EGFR), platelet-derived growth factor (PDGF), fibroblast growth factor (FGF), and insulin-like growth factor (IGF) via cyclooxygenase 2 (COX2) to promote tumor angiogenesis [31].

According to the results of GO and KEGG enrichment analysis, the targets of HDW's antiangiogenic effect in liver cancer are primarily involved in signalling pathways such as TNF-*α* and IL-17. TNF-*α* is a cytokine produced by macrophages or activated monocytes and plays a significant role in inflammatory response and tumor angiogenesis [32]. IL-17 is a highly versatile proinflammatory cytokine that has proven to be essential for tumor angiogenesis [33, 34]. It can upregulate IL-6 expression and activate the STAT3 pathway to exert protumour effects [35].

This research adopts the in vivo experiment to further validate the pharmacodynamic effects of HDW on anti-liver cancer and angiogenesis. The result shows that the injection of HDW could significantly reduce the liver lesion area and the serum levels of IL-6, IL-17, TNF-*α*, and IL-1*β* in the orthotopic liver cancer mouse model, and these results were dose-dependent to some extent. It suggests that HDW may attenuate tumor angiogenesis by lowering the levels of inflammatory factors. However, the exact mechanism needs further study.

In addition, it was known that the phosphorylation of Akt1 and STAT3 regulates the transcription of HIF-1*α*, a core molecule that induces tumor angiogenesis [36–38]. Phosphorylated Akt1 activates mTOR, which enhances HIF-1*α* transcription. Similarly, phosphorylated STAT3 in the nucleus enhances HIF-1*α* transcription, which in turn promotes the transcription of several proangiogenic genes such as VEGF, ultimately contributing to tumor angiogenesis [37, 38]. By detecting the expression of key proteins related to signalling pathways in tumor tissue, we found that the expression levels of p-Akt1 and p-STAT3 in the HDW groups were significantly lower than those of the model group. These findings suggest that HDW may regulate HIF-1*α* via modulating the level of phosphorylated Akt1 and STAT3 in several key signalling pathways, which ultimately affect tumor angiogenesis.

However, the shortcomings of this study inevitably exist. Specifically, the limitations of this study are as follows: the selection of the database is not comprehensive enough, the positive control group is lacking, and the validation of in vivo targets is not deep enough. In the next study, these shortcomings need to be further improved.

## 5. Conclusion

In conclusion, HDW may affect key targets such as Akt1, HIF-1, IL6, and IL-1*β*, as well as signalling pathways such as IL-17 and TNF-*α* via key active components such as DMQ, MMA, HHTCA, poriferasterol, stigmasterol, *β*-sitosterol and quercetin. It reflects the antitumor and antiangiogenesis effects of HDW that are multicomponent, multitarget, and multipathway. The result of the in vivo experiment further confirms the antiangiogenic effect of HDW, and it is supposed that its mechanism may be related to the inhibition of inflammatory factor production, which affects Akt1 and STAT3 phosphorylation, providing a reference for future studies on HDW's antiangiogenic mechanism.

## Figures and Tables

**Table 1:** A list of the final selected active compounds of HDW.

Molecule ID	Molecule name	OB (%)	DL
MOL001646	2,3-dimethoxy-6-methyanthraquinone	34.86	0.26
MOL001659	Poriferasterol	43.83	0.76
MOL001663	(4aS,6aR,6aS,6bR,8aR,10R,12aR,14bS)-10-hydroxy-2,2,6a,6b,9,9,12a-heptamethyl-1,3,4,5,6,6a,7,8,8a,10,11,12,13,14b-tetradecahydropicene-4a-carboxylic acid	32.03	0.76
MOL001670	2-Methoxy-3-methyl-9,10-anthraquinone	37.83	0.21
MOL000449	Stigmasterol	43.83	0.76
MOL000358	*β*-Sitosterol	36.91	0.75
MOL000098	Quercetin	46.43	0.28

## Data Availability

The data used to support the findings of this study are available from the corresponding author upon request.
